# Comparison of compressed sensing-sensitivity encoding (CS-SENSE) accelerated 3D T2W TSE sequence versus conventional 3D and 2D T2W TSE sequences in rectal cancer: a prospective study

**DOI:** 10.1007/s00261-022-03636-9

**Published:** 2022-08-23

**Authors:** Xiaoling Gong, Daguang Wen, Hong Wei, Yu Shen, Yujiao Deng, Ya Wang, Mingtian Wei, Xiaoxiao Zhang, Xiaoyong Zhang, Ziqiang Wang, Bing Wu

**Affiliations:** 1 Department of Radiology, West China Hospital of Sichuan University, No. 37, Guoxue Alley, Chengdu, 610041 Sichuan Province People’s Republic of China; 2 Department of Gastrointestinal Surgery, West China Hospital, Sichuan University, Chengdu, 610041 People’s Republic of China; 3 Philips Healthcare, Wuhan, China

**Keywords:** Compressed sensing-sensitivity encoding, Magnetic resonance imaging, Rectal neoplasms, Three-dimensional

## Abstract

**Purpose:**

This study aimed to evaluate the image quality and diagnostic value of compressed sensing-sensitivity encoding (CS-SENSE) accelerated 3-dimensional (3D) T2-weighted turbo spin-echo (T2W TSE) sequence in patients with rectal cancer compared with conventional 3D and 2-dimensional (2D) sequences.

**Methods:**

A total of 54 patients who underwent the above three sequences were enrolled. Two radiologists independently reviewed the image quality using an ordinal 5-point Likert scale. The quantitative measurement was performed to calculate the signal-to-noise ratio (SNR) and contrast-to-noise ratio (CNR). The diagnostic value was assessed using TN staging, extramural vascular invasion and mesorectal fascia status. Friedman and McNemar’s tests were applied for comparative analysis.

**Results:**

Forty-two patients were successfully included. Compared with 3D and 2D sequences, the CS-SENSE 3D sequence speeded up by 39% and 23%, respectively. The edge sharpness of CS-SENSE 3D images was similar to that of 3D and 2D images. The noise of CS-SENSE 3D images was comparable to that of 3D images but higher than that of 2D images. The SNR_tumor_ and SNR_rectal wall_ of CS-SENSE 3D images were considerably lower than those of 3D and 2D images. The CNR of CS-SENSE 3D images was similar to that of 3D images but lower than that of 2D images. However, no considerable differences were noted in diagnostic value among the three sequences.

**Conclusions:**

CS-SENSE 3D T2 sequence provided comparable diagnostic performance, with substantially reduced imaging time and no significant sacrifices in image quality. This technique may serve as a reliable tool for evaluating rectal cancer.

**Graphical abstract:**

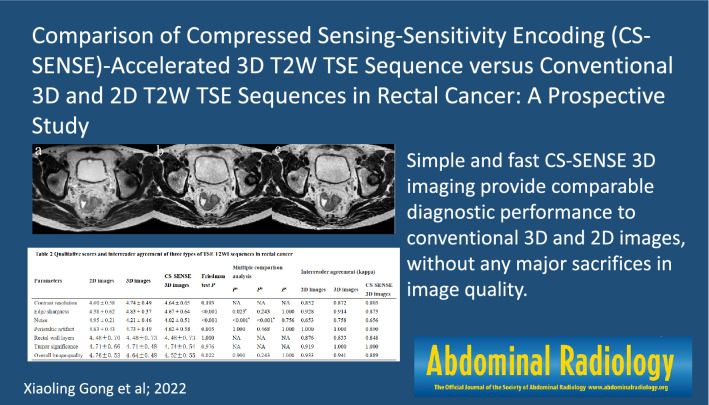

## Introduction

Colorectal carcinoma ranks the third most frequent malignancy and the second most common cause of cancer-related death globally [1]. High-resolution magnetic resonance imaging (MRI) can help diagnose T and N staging, extramural vascular infiltration (EMVI), and mesorectal fascia (MRF) status of rectal cancer. These are closely related to curative effect and prognosis and help in formulating individualized treatment plans and evaluating the effects of neoadjuvant therapy [2].

Multiplanar 2-dimensional (2D) high-resolution (HR) turbo spin-echo (TSE) T2-weighted imaging (T2WI) has excellent soft-tissue contrast and high spatial resolution and involves no radiation. It can help diagnose the T and N staging, EMVI, and MRF status, and thus has become one of the essential sequences in rectal MRI [3]. However, it still has a few limitations. For example, multiple-plane images obtained separately result in a relatively long total acquisition time. In addition, when the orthogonal-axis plane angle is improperly selected, 2D images can’t be reconstructed retrospectively due to their voxel anisotropy. Meanwhile, T staging may be overestimated because of the partial volume effect caused by the relatively thick slices [4]. Therefore, exploring a more simple, rapid, and high-quality MRI method is essential to guide the accurate diagnosis and treatment of rectal cancer.

Three-dimensional (3D) MRI can obtain isotropic data with high spatial resolution in a single scan and has the potential for multiplanar reconstruction (MPR). However, its clinical application is still restricted by the long scanning time and unclear diagnostic benefits [5]. The key to fast 3D MRI is mainly to remove the spatial and temporal redundancy during acquisition and reconstruction. Several acceleration technologies have been developed, including various parallel imaging technologies [6–8] and compressed sensing (CS) technology [9]. CS uses sparse characteristics of the original signal to collect discrete signal samples randomly. The imaging time is considerably shortened due to undersampling. At the same time, similar image quality can be maintained by reconstructing the signal using the nonlinear iterative reconstruction algorithm [8, 10]. Sensitivity encoding (SENSE) reduces scanning time using multiple receiver coils in parallel [7]. The 2 technologies accelerate imaging based on various principles, and hence it is theoretically practicable to combine them to reduce time further.

Compressed sensing-sensitivity encoding (CS-SENSE) is a combined method first proposed in 2009 [11]. It has been proved to be feasible in MRI using varying positions and sequences, such as T1 and T2 fluid-attenuated inversion recovery sequences in brain tumors, cine balanced steady-state free precession in structurally normal hearts, modified Dixon 3D gradient recalled echo in the hepatobiliary phase of gadoxetic acid-enhanced liver MRI in solid focal liver lesions, and intermediate-weighted sequences with fat saturation as well as T2- and T1-weighted sequences in ankles [12–15]. This technology provides a new approach for fast 3D imaging in rectal cancer. However, no relevant research has been reported so far. Therefore, our study aimed to evaluate the image quality and diagnostic value of CS-SENSE-accelerated 3D TSE T2WI sequence in patients with rectal cancer compared with conventional 2D and 3D TSE T2WI sequences.

## Materials and methods

### Patients

**Fig. 1:**
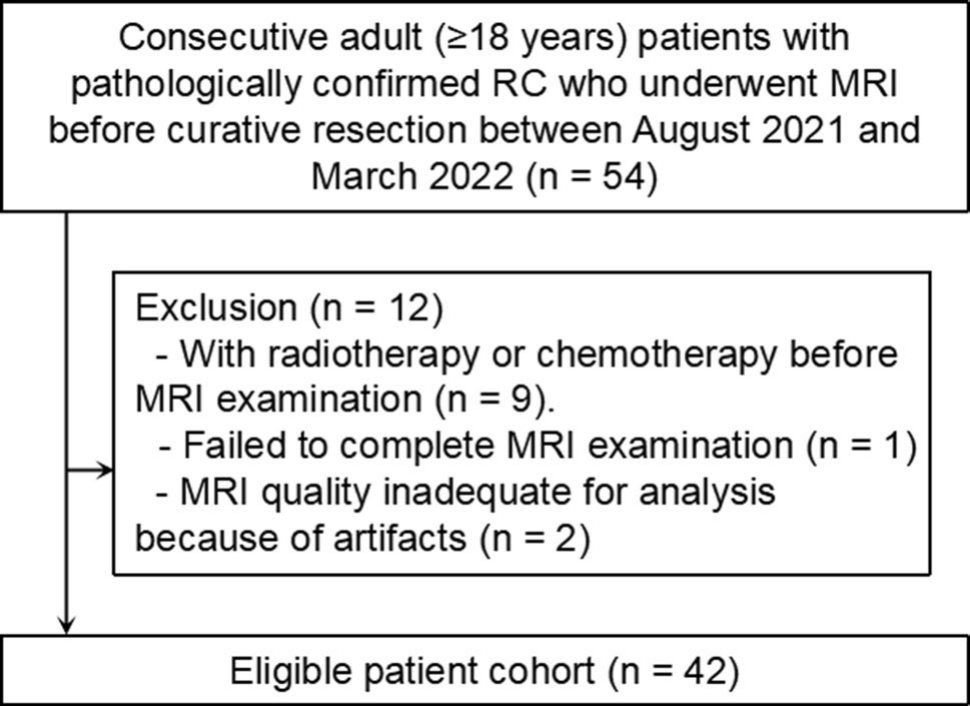
Flow diagram of participant selection in the study. *RC* rectal cancer, *MRI* magnetic resonance imaging

This prospective study was conducted following the ethical guidelines of the Declaration of Helsinki and approved by our Institutional Review Board. Written informed consent was obtained from all participants. Consecutive patients with biopsy-proven rectal cancer were prospectively recruited for the study. The exclusion criteria for recruitment were as follows: (a) contraindications to MRI examination, (b) incomplete MRI images, (c) MRI quality inadequate for analysis. The flow diagram of participant selection is depicted in Fig. 1.

### MRI protocols

All MRI examinations were performed on a 3.0-T system (Elition, Philips Healthcare, Best, the Netherlands) equipped with a 32-channel body coil combined with a 16-channel spine coil in the supine position. Raceanisodamine, 10 mg (Raceanisodamine Hydrochloride Injection, Minsheng, Hangzhou, China), was intramuscularly injected 10 min prior to the MRI examination to relieve intestinal peristalsis and took effect 1–2 min later, with a half-life of about 40 min. Two-dimensional sagittal images were first acquired to plan the axis of other images so that these images were oriented perpendicular to or parallel to the long axis of the segment of the rectum bearing the tumor. After the 2D images, 3D and CS-SENSE 3D images were collected. The details of MRI parameters are provided in Table 1.

**Table 1:** Imaging protocols for 3 types of T2WI sequences in rectal cancer

Parameters	2D TSE			3D TSE	CS-SENSE 3D TSE
Orientation	Sagittal	Oblique axial	Oblique coronal	Oblique axial	Oblique axial
Repetition time (ms)	4445	4167	4794	1250	1250
Echo time (ms)	110	100	100	148	148
TSE factor	25	25	25	120	120
Flip angle (degree)	90	90	90	90	90
Field of view (mm)	240 × 240 × 79	260 × 260 × 96	260 × 260	300 × 422 × 180	300 × 422 × 180
Matrix	300 × 252 × 24	324 × 299 × 48	324 × 307 × 32	376 × 519 × 450	376 × 519 × 450
Slice thickness/gap (mm)	3/0.3	3/0.8	3/0	0.8/–0.4	0.8/–0.4
Voxel dimensions (mm)	0.8 × 0.8 × 3.0	0.8 × 0.8 × 3.0	0.8 × 0.8 × 3.0	0.8 × 0.81 × 0.8	0.8 × 0.81 × 0.8
Voxel volume (mm^3^)	1.92	1.92	1.92	0.5184	0.5184
Number of slices	24	48	32	450	450
Phase sampling (%)	84	92.4	94.7	98.1	98.2
SENSE factor	2	2	2	2	NA
CS-SENSE factor	NA	NA	NA	NA	10
In-plane frequency encoding direction	Right-to-left	Right-to-left	Right-to-left	Right-to-left	Right-to-left
Acquisition time	2 min 4 s	4 min 43 s	1 min 46 s	10 min 43 s	6 min 33 s

*CS-SENSE* compressed sensing-sensitivity encoding, *NA* not applicable, *2D* two-dimensional, *3D* three-dimensional, *TSE* turbo spin echo, *T2WI* T2-weighted imaging

### Imaging analysis

All MRI images were evaluated using IntelliSpace Portal 10 (Philips Healthcare, the Netherlands). Two abdominal radiologists (G.X.L. and W.D.G., with 3 and 6 years of experience in gastrointestinal MRI, respectively), who were aware of the diagnosis of rectal carcinoma but blinded to other information, independently and successively performed the image analysis of 2D, 3D and CS-SENSE 3D data sets with at least a 4-week interval between analyses. Another reader (W.B., with over 20-years’ experience in abdominal MRI), aware of the diagnosis of rectal carcinoma but blinded to other information, pre-specified the slices to be measured during quantitative evaluation and ensured that they were the same among the three sequences. Readers could freely conduct multiplanar reconstruction in arbitrary orientations during their reads.

### Qualitative image analysis

The 2 reviewers independently assessed the contrast resolution, edge sharpness, noise, peristaltic artifact, tumor significance, visualization of rectal wall layers, and overall image quality of all images using an ordinal 5-point Likert scale (1 = poor; 2 = below average; 3 = adequate; 4 = good; and 5 = excellent), as previously reported [16]. An improved score indicated a higher image quality.

### Quantitative image analysis

The ROI of the tumor (ROI_tumor_) was drawn along the edge of the lesion on the slice with the largest tumor area. The ROI of the rectal wall (ROI_rectal wall_) was drawn along its inner and outer edges on the distal side of the high tumors (10.1–15.0 cm from the anal verge) or the proximal side of the low tumors (0–5.0 cm from the anal verge) or on either side of the mid tumors (5.1–10.0 cm from the anal verge) [17]. A circular ROI with an area of 100 mm^2^ was drawn on the obturator muscle (ROI_obturator muscle_) [18]. The mean signal intensity (SI) of the tumor and rectal wall and the standard deviation (SD) of the signal of the obturator muscle were measured. The signal-to-noise ratio (SNR) and contrast-to-noise ratio (CNR) were calculated as follows:

$$ {\text{SNR}}_{{{\text{tumor}}}} = \frac{{{\text{SI}}_{{{\text{tumor}}}} }}{{{\text{SD}}_{{{\text{obturator}}\,{\text{muscle}}}} }} $$

$$ {\text{SNR}}_{{{\text{rectal}}\,{\text{wall}}}} = \frac{{{\text{SI}}_{{{\text{rectal}}\,{\text{wall}}}} }}{{{\text{SD}}_{{{\text{obturator}}\,{\text{muscle}}}} }} $$

$$ {\text{CNR}} = \left| {{\text{SNR}}_{{{\text{tumor}}}} - {\text{SNR}}_{{{\text{rectal}}\,{\text{wall}}}} } \right| $$

### Diagnostic value analysis

T staging and N staging were based on the 7th edition of the American Joint Committee on Cancer (AJCC) cancer staging manual [19]. T1: extension to submucosa; T2: extension to muscularis propria; T3: extension to perirectal tissue (T3a: < 1 mm, T3b: 1–5 mm, T3c: 5–15 mm, T3d: > 15 mm); T4: perforation into visceral peritoneum (a) or invasion to other organs (b). N0: no involved regional nodes; N1: 1–3 involved regional nodes (N1a:1 involved lymph node; N1b: 2–3 involved lymph nodes; N1c: small deposits in the fat); N2: 4–6 involved lymph nodes (a) or 7 or more involved lymph nodes (b). The diagnostic criteria of positive lymph nodes included: (1) short axis diameter < 5 mm and 3 morphologically changes (round shape, irregular border and heterogeneous signal). (2) Short axis diameter 5–8 mm and ≥ 2 morphologically changes. (3) Short axis diameter ≥ 9 mm [3]. EMVI on MRI was defined as the irregular shape of the extramural vessels, focal enlargement of the vessel, and/or signal intensity of the tumor within the vessel [20]. Positive MRF was defined as the closest distance between the tumor and the mesorectal fascia less than or equal to 1 mm [21].

### Statistical analysis

All numeric values were recorded as the mean ± standard deviation. The Friedman test was performed to compare quantitative values, qualitative scores and the diagnosis of TN staging among the three protocols. Multiple comparative analyses were carried out when these results displayed statistical differences. The McNemar’s test was performed to compare the diagnosis of EMVI and MRF status among the three protocols. The interobserver agreement of qualitative evaluation and the diagnostic value analysis were evaluated using weighted kappa (≦0.1, poor; 0.1 < *k* ≤ 0.2, slight; 0.2 < *k* ≤ 0.4, fair; 0.4 < *k* ≤ 0.6, moderate; 0.6 < *k* ≤ 0.8, substantial; and 0.8 < *k* ≤ 1.0, almost perfect) [22]. The interobserver agreement of quantitative evaluation was evaluated using intraclass correlation coefficient values (0.00–0.39, poor agreement; 0.40–0.59, fair agreement; 0.60–0.74, good agreement; and 0.75–1.00, excellent agreement) [23]. The statistical analyses were performed with SPSS 26.0 (IBM, NY, USA). A 2-side *P* value < 0.05 indicated a statistically significant difference. If any multiple comparisons were performed, the *P* value was adjusted by the Bonferroni test.

## Results

### Study participants

Between August 2021 and March 2022, 42 consecutive patients (31 men and 11 women; mean age 56.57 ± 12.96 years, range 23–88 years) with biopsy-proven rectal cancer [distance from the anal verge 0–5.0 cm (9, 21%), 5.1–10.0 cm (28, 67%), and 10.1–15.0 cm (5, 12%), range 1.0–12.0 cm], who underwent MRI in our institution, were prospectively recruited for the study.

### Imaging time

CS-SENSE 3D sequence (6 min and 33 s) reduced the imaging time by 39% and 23%, respectively, compared with 3D (10 min and 43 s) and 2D sequences (8 min and 33 s).

### Qualitative image assessment

**Fig. 2:**
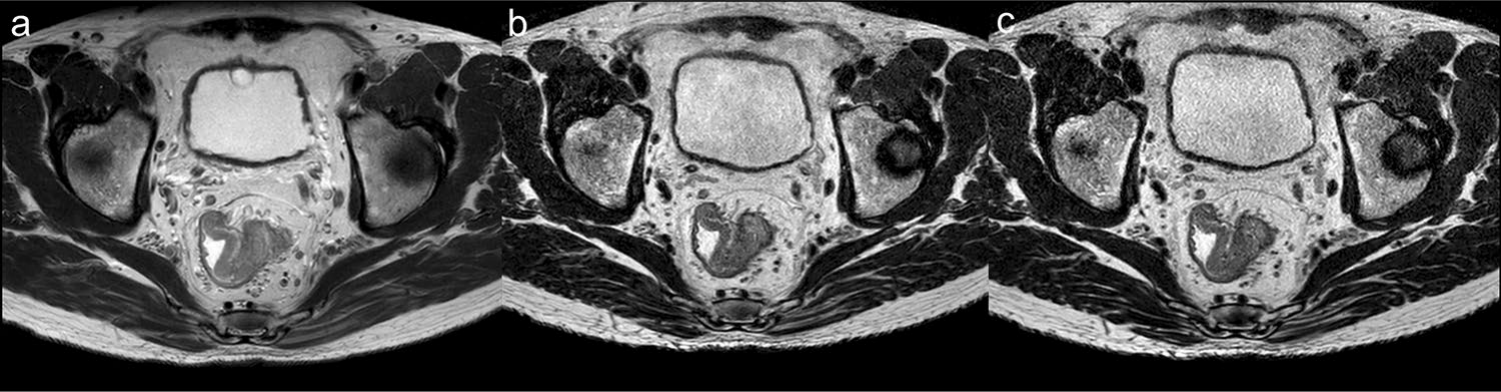
A 68-year-old male patient with rectal cancer. The scores of overall image quality on **a** 2D image, **b** 3D image and **c** CS-SENSE 3D image were 5, 4 and 4, respectively. The edge sharpness on CS-SENSE 3D image was similar to that on 2D image and 3D image. The noise on CS-SENSE 3D image and 3D image was similar, but they were more evident than the noise on 2D image. *CS-SENSE* compressed sensing-sensitivity encoding, *2D* two-dimensional, *3D* three-dimensional

The qualitative scores and interobserver agreement are summarized in Table 2. The edge sharpness of 3D images (4.83 ± 0.37) was remarkably superior to that of 2D images (4.38 ± 0.62) (*P* value = 0.023), whereas the edge sharpness of CS-SENSE 3D images (4.67 ± 0.64) was similar to that of 2D or 3D images (Fig. 2). The noise of CS-SENSE 3D images (4.02 ± 0.51) and 3D images (4.21 ± 0.46) was similar, but the noise of these 2 types of images was more obvious than the noise of 2D images (4.95 ± 0.21; both *P* values < 0.001; Fig. 2). No differences were observed in other image quality parameters among the 3 protocols.

**Table 2:** Qualitative scores and interreader agreement of three types of TSE T2WI sequences in rectal cancer

Parameters	2D images	3D images	CS-SENSE 3D images	Friedman test *P*	Multiple comparison analysis			Interreader agreement (kappa)		
					*P*^a^	*P*^b^	*P*^c^	2D images	3D images	CS-SENSE 3D images
Contrast resolution	4.60 ± 0.58	4.74 ± 0.49	4.64 ± 0.65	0.193	NA	NA	NA	0.852	0.872	0.803
Edge sharpness	4.38 ± 0.62	4.83 ± 0.37	4.67 ± 0.64	< 0.001	0.023*	0.243	1.000	0.928	0.914	0.873
Noise	4.95 ± 0.21	4.21 ± 0.46	4.02 ± 0.51	< 0.001	< 0.001*	< 0.001*	0.756	0.653	0.758	0.656
Peristaltic artifact	4.83 ± 0.43	4.73 ± 0.49	4.62 ± 0.58	0.005	1.000	0.468	1.000	1.000	1.000	0.890
Rectal wall layers	4.48 ± 0.70	4.48 ± 0.73	4.48 ± 0.73	1.000	NA	NA	NA	0.876	0.833	0.848
Tumor significance	4.71 ± 0.66	4.71 ± 0.48	4.74 ± 0.54	0.976	NA	NA	NA	0.919	1.000	1.000
Overall image quality	4.76 ± 0.53	4.64 ± 0.48	4.52 ± 0.55	0.022	0.900	0.243	1.000	0.933	0.941	0.889

Values are mean ± standard deviation. Significance values were adjusted by the Bonferroni correction for multiple tests. *P* < 0.05 indicated a statistically significant difference. The *P*^a^ value is for the comparison between 2D images and 3D images, *P*^b^ value is for the comparison between 2D images and CS-SENSE 3D images, and *P*^c^ value is for the comparison between 3D images and CS-SENSE 3D images

*CS-SENSE* compressed sensing-sensitivity encoding, *NA* not applicable, *2D* two-dimensional, *3D* three-dimensional, *TSE* turbo spin echo, *T2WI* T2-weighted imaging

*Statistically significant difference

### Quantitative image assessment

The quantitative data and interobserver agreement are summarized in Table 3. The SNR_tumor_, SNR_rectal wall_ and CNR were higher on 2D images (16.62 ± 3.19, 18.49 ± 3.32 and 2.53 ± 2.01, respectively) than on 3D images (7.07 ± 1.22, 8.56 ± 1.63 and 1.49 ± 1.11, respectively) and CS-SENSE 3D images (5.68 ± 1.00, 7.05 ± 1.61 and 1.39 ± 1.03, respectively). Also, the SNR_tumor_ and SNR_rectal wall_ were higher on 3D images than on CS-SENSE 3D images (all *P* values < 0.05) (Fig. 2).

**Table 3:** Quantitative evaluation and interreader agreement of three types of T2WI sequences in rectal cancer

Parameters	2D images	3D images	CS-SENSE 3D images	Friedman test *P*	Multiple comparison analysis			ICC (95% CI)		
					*P*^a^	*P*^b^	*P*^c^	2D images	3D images	CS-SENSE 3D images
SNR_tumor_	16.62 ± 3.19	7.07 ± 1.22	5.68 ± 1.00	< 0.001	< 0.001*	< 0.001*	< 0.001*	0.948 (0.905–0.972)	0.971 (0.946–0.977)	0.957 (0.921–0.977)
SNR_rectal wall_	18.49 ± 3.32	8.56 ± 1.63	7.05 ± 1.61	< 0.001	< 0.001*	< 0.001*	0.004*	0.964 (0.934–0.981)	0.876 (0.778–0.932)	0.967 (0.938–0.982)
CNR	2.53 ± 2.01	1.49 ± 1.11	1.39 ± 1.03	0.016	0.033*	0.045*	1.000	0.802 (0.659–0.890)	0.808 (0.663–0.893)	0.917 (0.826–0.958)

Values are mean ± standard deviation. Significance values were adjusted by the Bonferroni correction for multiple tests. *P* < 0.05 indicated a statistically significant difference. The *P*^a^ value is for the comparison between 2D images and 3D images, *P*^b^ value is for the comparison between 2D images and CS-SENSE 3D images, and *P*^c^ value is for the comparison between 3D images and CS-SENSE 3D images

*95% CI* 95% confidence interval, *CNR* contrast-to-noise ratio, *CS-SENSE* compressed sensing-sensitivity encoding, *ICC* intraclass correlation coefficient, *SNR* signal-to-noise ratio, *2D* two-dimensional, *3D* three-dimensional, *T2WI* T2-weighted imaging

*Statistically significant difference

### Diagnostic value assessment

**Fig. 3:**
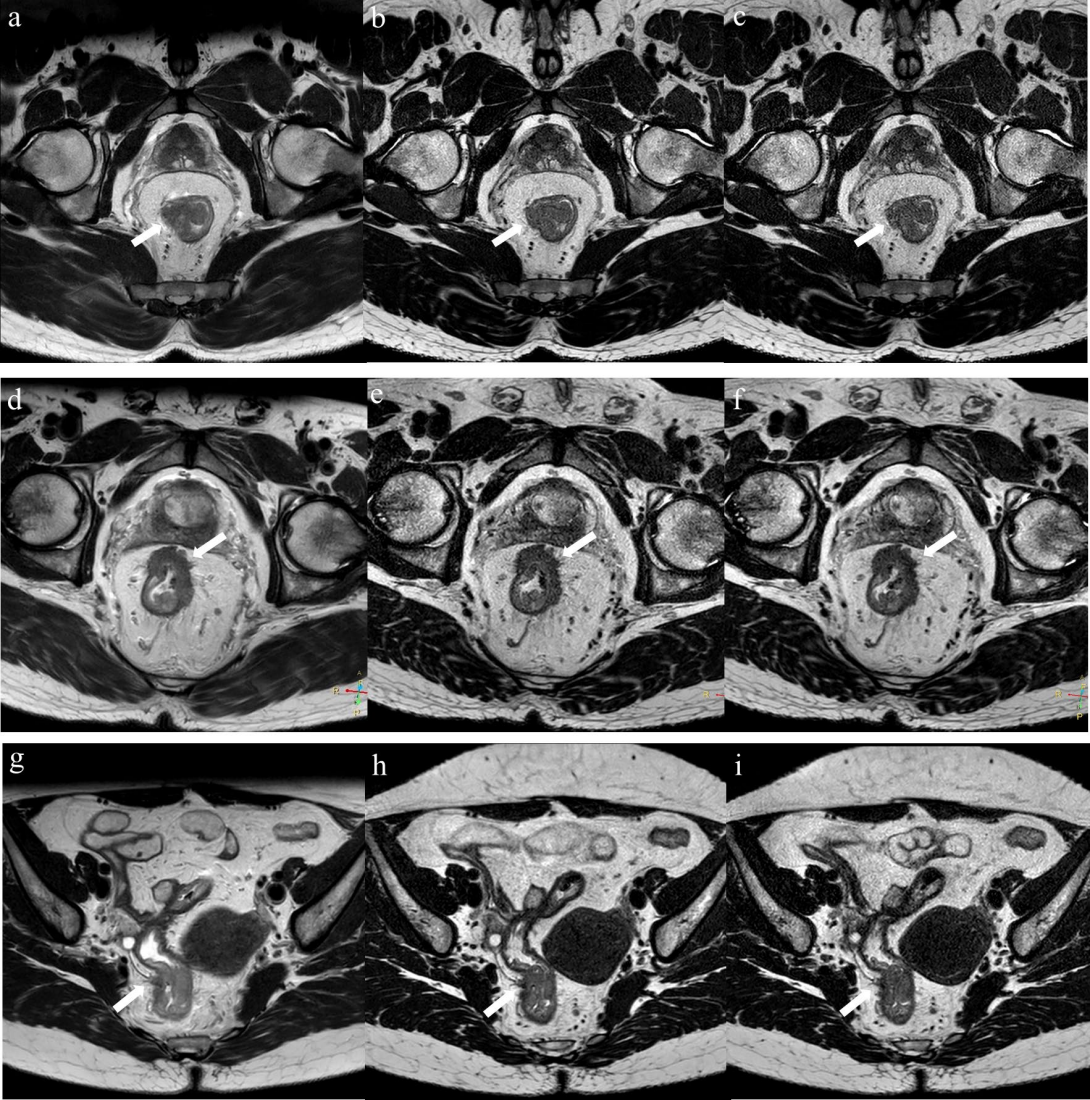
White arrows indicate the T staging of rectal cancers. **a**–**c** A T2 rectal cancer of a 61-year-old man on the 2D, 3D and CS-SENSE 3D images, respectively. **d**–**f** A T3 rectal cancer of a 50-year-old man on the 2D, 3D and CS-SENSE 3D images, respectively. **g**–**i** A T4 rectal cancer of a 48-year-old woman on the 2D, 3D and CS-SENSE 3D images, respectively. *CS-SENSE* compressed sensing-sensitivity encoding, *2D* two-dimensional, *3D* three-dimensional

The diagnostic capability and interreader agreement are summarized in Tables 4 and 5. Although 2D images might cause over-staging of T2 tumors (Fig. 3) due to the partial volume effects, there was no statistical difference in the diagnostic value of 2D, 3D and CS-SENSE 3D sequences for T staging in our study. No remarkable differences were observed among the 3 sequences in the diagnosis of N staging, EMVI, and MRF status as well.

**Table 4:** Diagnostic capability and interreader assessment for three TSE T2WI sequences in rectal cancer

Parameters	2D images		3D images		CS-SENSE 3D images		Friedman test *P*	Multiple comparison analysis			Interreader agreement (kappa)		
	Reader 1	Reader 2	Reader 1	Reader 2	Reader 1	Reader 2		*P*^a^	*P*^b^	*P*^c^	2D images	3D images	CS-SENSE 3D images
mrT1-2^a^	33	33	35	35	35	35	0.905	NA	NA	NA	0.921	1.000	0.957
mrT3a	2	0	0	0	0	0							
mrT3b	4	6	4	4	4	4							
mrT3c	2	2	2	2	2	2							
mrT3d	0	0	0	0	0	0							
mrT4a	1	1	1	1	1	1							
mrT4b	0	0	0	0	0	0							
mrN0	34	34	34	34	34	34	1.000	NA	NA	NA	1.000	1.000	0.941
mrN1a	4	4	4	4	4	4							
mrN1b	4	4	4	4	4	4							
mrN1c	0	0	0	0	0	0							
mrN2a	0	0	0	0	0	0							
mrN2b	0	0	0	0	0	0							

Unless otherwise indicated, data are numbers of patients, with percentages in parentheses. Significance values were adjusted by the Bonferroni correction for multiple tests. *P* < 0.05 indicated a statistically significant difference. The *P*^a^ value is for the comparison between 2D images and 3D images, *P*^b^ value is for the comparison between 2D images and CS-SENSE 3D images, and *P*^c^ value is for the comparison between 3D images and CS-SENSE 3D images

*CS-SENSE* compressed sensing-sensitivity encoding, *NA* not applicable, *2D* two-dimensional, *3D* three-dimensional, *TSE* turbo spin echo, *T2WI* T2-weighted imaging

^a^MRI has limited ability to distinguish T1 and T2 tumors

**Table 5:** Diagnostic capability and interreader assessment for three TSE T2WI sequences in rectal cancer

Parameters	2D images		3D images		CS-SENSE 3D images		McNemar test *P*			Interreader agreement (kappa)		
	Reader 1	Reader 2	Reader 1	Reader 2	Reader 1	Reader 2	*P*^a^	*P*^b^	*P*^c^	2D images	3D images	CS-SENSE 3D images
mrEMVI (−)	40	39	39	39	39	39	0.333	0.333	0.333	0.788	1.000	1.000
mrEMVI (+)	2	3	3	3	3	3						
mrMRF (−)	39	39	39	39	39	39	0.333	0.333	0.333	1.000	1.000	1.000
mrMRF (+)	3	3	3	3	3	3						

Unless otherwise indicated, data are numbers of patients, with percentages in parentheses. Significance values were adjusted by the Bonferroni correction for multiple tests. *P* < 0.05 indicated a statistically significant difference. The *P*^a^ value is for the comparison between 2D images and 3D images, *P*^b^ value is for the comparison between 2D images and CS-SENSE 3D images, and *P*^c^ value is for the comparison between 3D images and CS-SENSE 3D images

*CS-SENSE* compressed sensing-sensitivity encoding, *NA* not applicable, *2D* two-dimensional, *3D* three-dimensional, *TSE* turbo spin echo, *T2WI* T2-weighted imaging, *EMVI* extramural vascular infiltration, *MRF* mesorectal fascia

## Discussion

Our study demonstrated that CS-SENSE 3D sequence substantially reduced the imaging time without any major sacrifices in image quality and provided comparable image quality and diagnostic accuracy to both 3D and 2D images.

In terms of shortening imaging time while maintaining image quality by using CS-SENSE in 3D imaging, our study was in agreement with several previous studies of CS-SENSE in the imaging of the liver, pancreaticobiliary duct, brain, vessels, and musculoskeletal system [14, 15, 24–28]. The acceleration factor of CS-SENSE in the present study was 10, which provided balanced image quality and acquisition time. Increasing the CS-SENSE factor could further reduce the acquisition time; however, the image quality might be challenged by the higher acceleration factor. Fortunately, coordinating the acceleration factor with other imaging parameters, improving the reconstruction algorithm, and developing artificial intelligence (AI) may allow further acceleration of 3D imaging in rectal cancer [29].

In the present study, the qualitative analysis revealed that the above-mentioned images presented almost the same degree of peristalsis artifact. We speculated that this might be related to the small proportion of high tumors in our study (5/42), which may risk underestimating peristaltic artifacts in CS-SENSE 3D and 3D images. Therefore, subsequent studies need to include more participants and involve subgroup analysis between the high-tumor group and the mid-tumor and low-tumor groups to determine the impact of the peristaltic artifact of CS-SENSE on image quality. Further, we demonstrated that the edge sharpness of 3D images outperformed that of 2D images, whereas the edge sharpness of CS-SENSE 3D images was similar to that of 2D images. This might be because the interlayer resolution was higher on 3D images than on 2D images, and CS-SENSE could denoise the images [30].

In the current study, the quantitative analysis revealed that the SNR_tumor_ and SNR_rectal wall_ decreased progressively from 2 to 3D to CS-SENSE 3D images and the CNRs of the tumor and rectal wall on 3D and CS-SENSE 3D images were similar but lower than those on the 2D images. This occurred because the small voxel size limited SNRs on the CS-SENSE 3D and 3D images due to the isotropic high spatial resolution of 3D images [18, 31]. When other imaging parameters remained unchanged, the undersampling led to a decline in SNR. This explained why the SNR was lower on CS-SENSE 3D images than on 3D images. Although our CS-SENSE 3D sequence already balanced denoise (system denoising = 15%), acceleration factor (CS-SENSE = 10), and image details, its noise was still more obvious than that of 2D images. Fortunately, it did not impact the diagnosis. Nevertheless, whether the SNR and CNR of CS-SENSE-accelerated images outperform those of the conventional images remained controversial in the prior work [15, 27, 28]. Additionally, our study found that the image noise on the same sequences with or without CS-SENSE acceleration would not be significantly different. This was inconsistent with the study by Nam et al. in which the noise on images accelerated by CS-SENSE was higher than that on images accelerated by SENSE [14]. The possible reasons for the discrepancy between our study and previous studies may be the different sequences, target organs and calculation formulas of SNR and CNR.

No remarkable differences were observed among CS-SENSE 3D images, 3D images, and 2D images in terms of the diagnosis of TN staging, EMVI, and MRF status in this study. This could be explained by the fact that the image quality of the 3 sequences was good or excellent. Additionally, the reduced SNR and CNR were insufficient to influence the diagnostic assessment.

Our study had several limitations. First, our study sample was small. Hence, the repeatability of CS-SENSE 3D imaging needs to be verified through multicenter research. Second, we did not compare the quality of sagittal and coronal images. Finally, we failed to confirm our findings by using the pathology as the gold standard, because the detailed data of pathological staging were unavailable. Future studies are warranted to evaluate the consistency between the preoperative MR findings and pathological results.

In conclusion, our study demonstrated that CS-SENSE 3D imaging could considerably simplify and speed up the MRI examination of rectal cancer without any major sacrifices in image quality, and provide comparable diagnostic performance to conventional 3D and 2D images. Therefore, CS-SENSE 3D imaging may be recommended to improve the workflow of rectal cancer.
